# ScaleDefrag: Design and implementation of a scalable file defragmentation tool for flash-based SSDs

**DOI:** 10.1371/journal.pone.0348520

**Published:** 2026-05-06

**Authors:** Sangjin Lee, Sunggon Kim, Yongseok Son

**Affiliations:** 1 Department of Computer Science and Engineering, Chung-Ang University, Seoul, Republic of Korea; 2 Department of Computer Science and Engineering, Seoul National University of Science and Technology, Seoul, Republic of Korea; University of Lagos Faculty of Engineering, NIGERIA

## Abstract

We propose ScaleDefrag, a parallel and asynchronous defragmentation tool that reduces defragmentation time by up to 3.8× compared to e4defrag, while improving scalability on multi-core systems. Flash-based solid-state drives (SSDs) have been widely adopted in various large-scale storage systems including cloud and HPC environments. Unfortunately, even with high-performance flash-based SSDs, intensive file modifications can provoke file fragmentation by scattering file data across non-contiguous blocks and splits large requests into several smaller I/O requests, which degrades performance. To address this fragmentation issue, the defragmentation technique gathers the scattered blocks into contiguous space. However, the current technique does not scale well since the defragmentation process is performed by a single defragger sequentially and synchronously. ScaleDefrag scales the defragmentation process as follows. Specifically, we first devise an information collector that gathers metadata for all target files before parallel defragmentation. Second, to realize parallel defragmentation, we devise multiple defraggers with the previously collected file information, enabling a one-to-one (defragger-to-file) model. Finally, we adopt an asynchronous I/O strategy to enable each defragger to issue multiple requests for the scattered blocks and complete them asynchronously. We implement ScaleDefrag and evaluate its performance on a multi-core machine with a flash-based SSD. Our evaluation results on a commodity SSD show that ScaleDefrag reduces defragmentation time and increases defragmentation throughput across various workloads, while also mitigating the slowdown experienced by a co-running application compared with e4defrag.

## Introduction

Flash-based SSDs have been widely used since they provide high throughput, low latency [1–4], and their own internal parallelism [5–9]. With these advantages, flash-based SSDs are actively adopted in various storage systems, including cloud computing [10], enterprise storage systems [11,12], machine-learning [13] and big data analytics [14], and graph analytic systems [15,16]. In such modern systems, it is common for multiple applications to perform concurrent I/O requests simultaneously. Unfortunately, extensive I/O and file creation/deletion from numerous applications can increase the chance of making fragmented files. Thus, the existing file systems such as EXT4, XFS, and F2FS can suffer from file fragmentation [17,18]. In a file fragmentation state, file data is scattered across multiple non-contiguous regions instead of a single contiguous one [19–21].

Studies about fragmentation have been widely conducted for many years due to its negative influence on I/O performance on storage devices [17,22–28]. The literatures show that, even though flash-based SSDs provide higher I/O performance than traditional HDD disks, SSD performance can be degraded due to file fragmentation [17,23,25,29,30]. Especially, a high degree of file fragmentation increases I/O latency and decreases I/O throughput because the file system should read several scattered blocks when reading a fragmented file and it cannot provide continuous free space for write requests [19,31], resulting in degradation of the storage stack performance. Specifically, file fragmentation can split a large single request into several smaller requests, increasing the number of I/O operations between the host and SSD [18]. Furthermore, flash-based SSDs have an internal buffer cache storing recent and/or commonly used data and mapping tables in the near future [32]. Thus, accessing highly fragmented files reduces the hit rate of the internal buffer cache of SSD.

Accordingly, the defragmentation technique is used on storage systems to handle these fragmentation issues. For example, e4defrag, an online defragmentation tool, for the EXT4 file system [33], reduces fragmentation by gathering the scattered blocks of a fragmented file into a newly allocated contiguous space. Through the defragmentation procedure, the files can be read or written by relatively fewer I/O requests (i.e., contiguous blocks), which improves the overall I/O performance [23,27]. Although the existing defragmentation technique improves the overall performance, it is based on HDDs and cannot fully exploit the characteristics of flash-based SSDs. Specifically, e4defrag performs the defragmentation process by a single defragger in a serialized manner, and the single defragger performs synchronous I/O operations when gathering scattered blocks of a fragmented file during the defragmentation process. As a result, these procedures make the storage maintenance process slow. Modern SSDs expose substantial internal parallelism, but reaching peak performance typically requires maintaining sufficient I/O concurrency (e.g., multiple outstanding requests). However, a single defragger and synchronous I/O limit the number of in-flight requests and underutilize both SSD parallelism and multi-core resources. Consequently, existing tools can prolong maintenance time and increase interference with co-running workloads.

To improve this defragmentation process, previous studies have investigated the defragmentation process for flash-based SSDs. As shown in Table 1, Park et al. [27] target a log-structured file system (LFS), Janusd [23] introduces a decoupled defragger that takes advantage of flash storage’s internal logical block to a physical block mapping table. It remaps the logical block addresses (LBAs) of the logically fragmented files with the Flash translation layer (FTL) mapping table, assuming SSDs expose the FTL mapping table to the host. Jun et al. [43] require NVMe command extensions and controller changes. FragPicker [21] minimizes the amount of I/Os during the defragmentation process by only migrating necessary blocks of files. As summarized in Table 1, these schemes either rely on non-commodity or modified hardware, or focus primarily on minimizing extra I/O traffic rather than exploiting parallelism. Moreover, they do not jointly exploit host multi-core parallelism and asynchronous I/O on commodity SSDs. Our study is in line with these studies [21,23] in terms of investigating file fragmentation and improving file system performance. In contrast, we focus on combining parallel defragmentation with asynchronous I/O to increase I/O concurrency and exploit the SSD internal parallelism while still leveraging off-the-shelf SSDs.

**Table 1:** Categories and comparison with previous defragmentation studies.

Scheme	EXT4 support	Commodity SSDs	Low extra I/Os	Parallelism
e4defrag [33]	✓	✓		
Park et al. [27]		✓		
Janusd [23]	✓		✓	
Jun et al. [43]	✓			
FragPicker [21]	✓	✓	✓	
**Our study** (ScaleDefrag)	✓	✓		✓

✓: supported; otherwise not supported or not a primary focus.

**EXT4 support**: runs on commodity EXT4; **Commodity SSDs**: works on off-the-shelf SSDs without FTL/NVMe/hardware changes; **Low extra I/Os**: reduces additional I/O traffic from defragmentation; **Parallelism**: exploits multi-core and/or SSD internal parallelism.

In this article, to reduce the defragmentation time, we propose a scalable file defragmentation tool called ScaleDefrag. Our goal is to leverage the internal parallelism of SSDs by parallel and concurrent I/O techniques [34–36]. To do this, first, we devise an information collector to collect the information of all target files before parallel defragmentation. Second, to realize parallel defragmentation, we devise multiple defraggers to perform defragmentation and I/O operations for fragmented files in parallel with the previously collected file information, enabling a one-to-one (defragger-to-file) model instead of a one-to-all (defragger-to-all files) model. This strategy can take advantage of the inherent parallelism of multi-cores and SSD by increasing the number of parallel I/O requests.

Finally, we adopt an asynchronous I/O strategy to enable each defragger to issue multiple requests of the scattered blocks of a fragmented file and complete them asynchronously. For example, each defragger issues all read requests of the scattered blocks, then, it waits and completes the issued read requests. This strategy can increase the number of concurrent I/O requests per defragger. We implement our ScaleDefrag with three techniques based on e4defrag [33,37] and evaluate it on a multi-core machine with a flash-based SSD. The experimental results show that ScaleDefrag reduces the execution time of defragmentation by up to 3.81× compared with the existing tool (e4defrag). Finally, we also open source code and it is available at https://github.com/syslab-CAU/ScaleDefrag to aid future fragmentation studies.

In our previous work [38], we introduce the information collector and parallel defragmentation. In this article, we extend our scheme by devising the asynchronous I/O technique. By combining asynchronous I/O and parallel defragmentation, ScaleDefrag performs I/O operations more efficiently regardless of individual request completion status during file defragmentation. Specifically, the parallel scheme alone achieves a 2.56× performance gain over the existing defragmentation scheme, while adding asynchronous I/O alongside the parallel scheme yields an additional 1.49× improvement over parallel only scheme. Consequently, ScaleDefrag gains total 3.81× of performance over the e4defrag, indicating that both parallel and asynchronous I/O schemes are essential components. As a result, our enhanced scheme further improves the defragmentation performance compared to our prior work [38]. We note that this article is a substantially extended journal version of our prior conference paper [38]. This article introduces the asynchronous I/O technique, provides background and design/implementation in more detail, includes a performance breakdown from the defragmentation application layer to the Linux kernel, extends the evaluations by adding various evaluation results of asynchronous technique, various macro-benchmarks, co-running impact, core scalability in manycores, and adds correctness/consistency, discussion/limitation, and related work sections.

The rest of the paper is organized as follows. Background and Motivation section introduces the background and motivation, and Design and Implementation section details the proposed design and implementation. Discussion and limitation section discusses limitations, Evaluation section presents the experimental results, Related work section reviews related work, and Conclusion section concludes the paper.

## Background and motivation

### File defragmentation on SSD

The nature of spinning disks in HDD restricts the performance of the storage system [39]. Moreover, in HDD-based storage systems, accessing a highly-fragmented file reduces the performance due to the increased time-consuming seek operations [23]. Thus it is well known that file fragmentation can severely degrade the performance of the entire system which is backed with a large volume of HDDs, and can worsen with frequent file I/O operations.

In contrast, some studies [40,41] have not recommended defragmentation for flash-based SSDs. Since flash-based SSDs do not require additional seek operations, it is believed that the effect of defragmentation on the I/O performance is rather negligible. However, previous literature [17,21,23,25,29] shows that the fragmentation can cause up to 5× degradation of I/O performance on SSDs.

The degradation of application performance on flash-based SSD by file fragmentation comes from two main reasons [21,25,27]. First, the file fragmentation can split a large request into several smaller requests which increases the number of I/O operations between the host and SSD. Second, modern commodity SSDs have an internal buffer cache, which is used to store user data and mapping tables [32]. SSDs internally read recently used data and adjacent data into the internal data cache via prefetching. Thus, accessing fragmented data within the file can diminish the benefits of prefetching, as data associated with other files may be prefetched and then evicted, reducing the cache hit ratio and spatial locality [42]. As a result, accessing highly fragmented files reduces the hit rate of the cache, which in turn hinders I/O performance [18,43].

**Fig 1:**
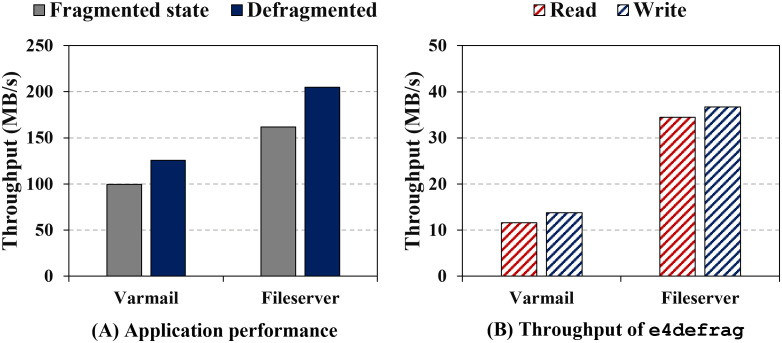
Motivational performance evaluation. **(Fragmented state: it indicates the throughput measured after file generation, before defragmentation. Defragmented: it indicates the throughput after running e4defrag on the same workload.).** (A) Application performance of varmail(highly fragmented) and fileserver(moderately fragmented) workloads in fragmented and defragmented states. (B) Read and Write throughput of **e4defrag** during defragmentation. Defragmentation increases application throughput while the e4defrag still underutilizes SSD bandwidth.

We conduct a motivational experiment to show how file fragmentation affects the realistic application performance on commodity SSD, as illustrated in Fig 1A. The details of the experimental setup are described in the Experimental setup subsection. For more realistic evaluations, we use two varmail and fileserver workloads in filebench [44]. The varmail workload makes SSD a high-fragmented state, and the fileserver workload makes a medium-fragmented state. According to the fragmentation score used in e4defrag, the varmail workload shows 84 points and the fileserver workload represents 27 points (detailed information on workloads can be found in Table 4.)

Then, we run each workload at fragmented and defragmented states. As shown in the figure, in the case of varmail and fileserver, the throughput at the defragmented states is higher by up to 26.5% and 27% than that of fragmented SSD, respectively. Consequently, the result shows that defragmentation can be effective and improve the application and I/O performance on flash-based SSDs.

### Under-utilization of SSD parallelism

Generally, flash-based SSDs are composed of a group of parallel I/O units. There are four different types of parallelisms inside SSDs: (1) channels (channel-level), (2) chips in a channel (chip-level), (3) dies in a chip (die-level), and (4) planes in a die (plane-level) [36,45]. Exploiting these internal parallelism of modern SSDs is the key to maximize the performance of SSDs [46–48]. Specifically, previous studies [34–36] show the optimal conditions and solutions to exploit the parallelism of SSD. For example, they demonstrate that increasing the number of parallel and concurrent I/O requests can maximize the utilization of SSD parallelism. Thus, the strategies of I/O operations by a single thread or a single/small request at a time can degrade the SSD performance.

As shown in Fig 1B, we observe the existing defragmentation scheme cannot exploit SSD parallelism and performance. We measure the I/O throughput of e4defrag during defragmentation on a SSD. The experimental setup and workloads are same as those in the evaluation shown in Fig 1A. The maximum throughput of e4defrag is around 14 MB/s and 36 MB/s in the case of varmail and fileserver, respectively. The flash-based SSD [49] we use can provide maximum random read and random write throughput up to 336 MB/s and 332 MB/s, respectively. The defragmentation process includes other operations (e.g., memory copy and computation) as well as I/O operations, it may not reach the peak performance of flash devices. However, we observe that the defragmentation process can still further utilize the performance and internal parallelism of SSDs.

### Procedures of defragmentation

Defragmentation reduces the degree of file fragmentation by gathering the scattered blocks of a file and relocating the blocks sequentially. The reducing file fragmentation degree can prevent additional undesired access incurred by highly dispersed I/O patterns and reduce overhead on the I/O stack by creating more contiguous free space in storage, increasing the spatial locality [25,43]. In this article, we analyze and focus on the defragmentation process of an existing defragmentation tool (i.e., e4defrag) of EXT4 file system since it is one of the most widely used file systems.

#### Serialized defragmentation process.

**Fig 2:**
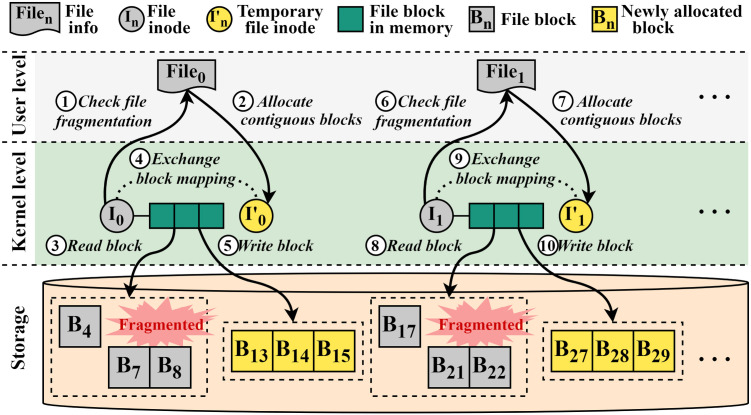
Overall procedure of serialized defragmentation process in the existing defragmentation scheme (e4defrag). The e4defrag processes files sequentially with a single defragger, limiting multi-core parallelism and I/O concurrency.

Fig 2 shows the overall procedure of e4defrag. As shown in the figure, the working procedure of e4defrag is performed in both user space and kernel space. In the user space, a single defragger (thread) gathers the file information of a target file (e.g., absolute file path, file size, the number of blocks of the file, etc.) by using its inode. Then, the defragger determines whether the file is fragmented or not by using the file information ①. If the file is not fragmented, the defragger gathers the file information of the following file. In the case of the fragmented file, the defragger calculates the fragmentation rate and creates a temporary file of the same size as the fragmented file by allocating a group of continuous free blocks ②. Then, the defragger starts to migrate the scattered blocks to the allocated continuous space by calling a special ioctl system call (i.e., EXT4_IOC_MOVE_EXT()).

Subsequently, the defragger reads scattered blocks (e.g., B_4_, B_7_, and B_8_) of the fragmented file (file_0_) from the storage to the memory (i.e., page cache) ③. Then, the defragger exchanges the block mapping information of the fragmented file (I_0_) and the temporary file (I_0_′) ④ and writes the file data to the allocated contiguous blocks (block_13_, block_14_, and block_15_) ⑤. The temporary file is unlinked after writing the data so that its related blocks (block_4_, block_7_, and block_8_) will be automatically reclaimed later by the file system. After the defragmentation of file_0_, the defragger starts working on next target file (file_1_) as the same procedure (⑥–⑩). This procedure shows that e4defrag performs the defragmentation process on the target files in a serialized manner with a single defragger thread.

#### Synchronous I/O.

The existing scheme performs a synchronous I/O operation when gathering scattered blocks during defragmentation. For example, when reading scattered blocks from storage to memory, the defragger thread issues one scattered block and completes the block one by one. This means that the next scattered block cannot be issued until the previously issued block is completed, resulting in underutilization of the storage resource.

**Fig 3:**
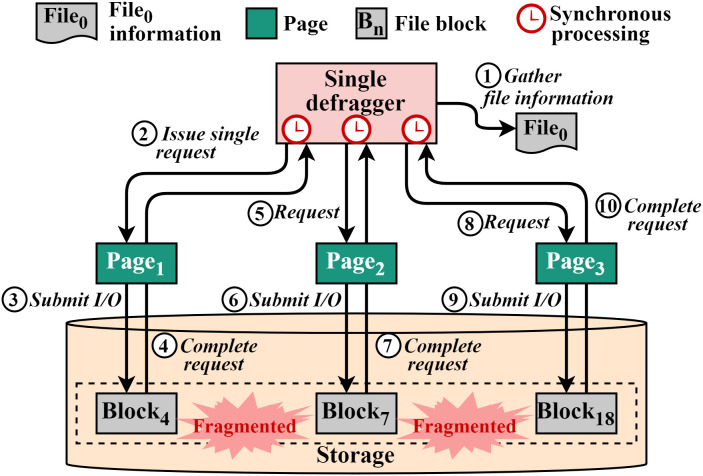
Synchronous I/O operation in the existing defragmentation tool (e4defrag). The defragger issues and completes scattered-block I/O ont at a time (i.e., at most one outstanding request), underutilizing SSD internal parallelism and increasing defragmentation time.

To provide a detailed explanation in e4defrag, Fig 3 shows a synchronous operation with a fragmented file that has non-contiguous blocks. First, the single defragger gathers file information to check and start defragmentation ①. After checking file information, including the fragmentation state, a defragger grabs a page (page_1_) for reading a block (block_4_) ②. Then, the defragger submits an I/O request with the page to the storage device ③ and waits for the completion. Finally, after the I/O request is completed, the defragger completes the I/O operation for the block ④. Note that the defragger can issue the next request (e.g., block_7_) only after the previous I/O operation is completely finished. Likewise, the blocks with the pages (e.g., page_2_ and page_3_) will be processed the same as the above. As a result, since there can only be one read request to the storage device at once, this approach cannot exploit the high parallelism of modern hardware (i.e., multi-cores and multi-channel SSDs), increasing the defragmentation time. To mitigate the problem, we adopt the asynchronous I/O scheme as presented in the Asynchronous I/O subsection.

## Design and Implementation

**Fig 4:**
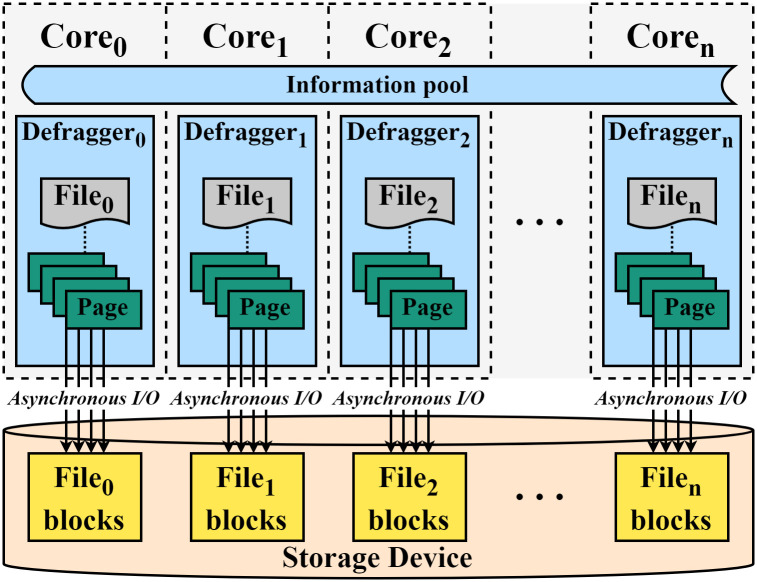
Overall Architecture of ScaleDefrag.

In this section, we present a scalable and asynchronous file defragmentation tool called ScaleDefrag to reduce the defragmentation time by leveraging the internal parallelism of multi-cores and SSDs [34–36] as shown in Fig 4. Specifically, we first identify fragmented files and collect their file information to prepare parallel defragmentation via a collector. Second, we devise multiple defraggers to perform the file defragmentation for different files in parallel with the previously collected information. This enables each defragger to handle each file individually, creating one-to-one (defragger-to-file) model instead of one-to-all (defragger-to-files) model as shown in Fig 4. As a result, it increases the number of parallel I/O requests. Finally, we use an asynchronous I/O approach that enables each defragger to issue all blocks of the target file, and after that, complete them asynchronously. This scheme increases the number of concurrent I/O requests per defragger. We implement ScaleDefrag based on the EXT4 file defragmentation tool, e4defrag [33]. We describe the details of ScaleDefrag in the following subsections.

### Collecting file information for parallelization

**File information collector:** A process of defragmentation can be performed in different scopes. For example, the process can occur for a file, a directory, or a device. If the requested unit to perform defragmentation is a directory, we need to identify all the regular files under the directory and then collect their information in advance (e.g., absolute file path, file size, the number of blocks of the file, etc.) for parallel processing later. To do this, we employ an information collector to traverse the given directory as a root and collect the information of the files under the directory. The collector traverses the directory tree of the file system in a breadth-first way. Whenever a directory is encountered, we enqueue it into a queue which is used for traversing the tree. If a regular file is encountered, the collector stores the information of the file in a file information pool. The file information pool will be used by multiple defraggers for parallel defragmentation. The information collector is single-threaded and appends file descriptors to the file information pool. After the collection completes, the pool becomes read-only (no further insertions or updates), so defraggers only perform concurrent reads of immutable entries. After all regular files in a directory are gathered, the information collector dequeues the next directory and starts to gather the file information in the directory. After the collector gathers all the information of the files in all directories under the given directory (i.e., root), the collector invokes multiple defraggers to start parallel defragmentation. The breadth-first method to traverse the directory tree allows defraggers to process files in the same directory first before processing files in another directory. As a result, files in the same directory are more likely to be placed in close to each other. This approach improves the spatial locality of data in the same directory, and may facilitate workloads such as reading all files under one directory. In addition, we check the file fragmentation after placing the files in the file information pool for parallel checking by multiple defraggers, instead of serializing the check operations by a single defragger. Furthermore, regardless of checking file fragmentation before or after information collection, the defragger may still end up processing a file that is not adjacent to others. This is because there is still a chance that files are selected non-sequentially due to skipped files in both cases.

**Fig 5:**
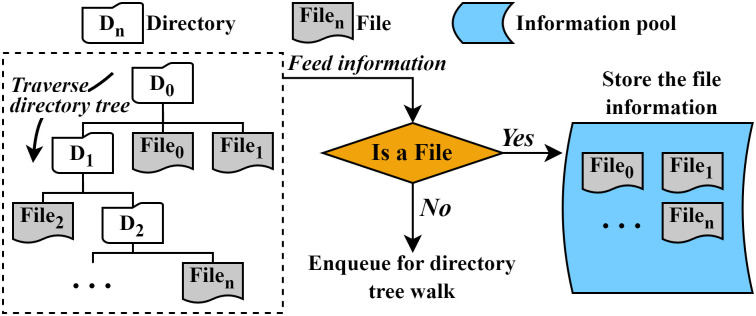
Overall process of file information collection in ScaleDefrag.

**Procedure:** Fig 5 shows the overall process of a collector on a root directory (D_0_) with N files. The collector traverses the files under the given directory. As shown in the figure, since the first entry (D_1_) is a directory, the collector enqueues the entry into a queue and checks the next entry. Since the second entry and the third entry (file_0_, file_1_) are regular files, the collector stores the file information in the file information pool. When all the entries under D_0_ are checked, the collector dequeues a directory from the queue, which is D_1_, and checks the entries in that directory in the same way. The collector traverses all remaining entries recursively (file_2_-file_*n*_) until all the entries under the root directory are checked. Afterward, the collector invokes multiple defraggers. The defraggers start to fetch file information from the information pool in a FIFO manner and perform defragmentation tasks.

### Parallel defragmentation

As described in the Procedures of defragmentation subsection, the existing e4defrag performs the single-threaded defragmentation process in a serialized manner. This scheme cannot fully exploit the parallelism of SSD since a single defragger processes all the files and issues a single request at a time. To increase the number of parallel I/O requests, we present multiple defraggers to enable the one-to-one model where each defragger defragments its allocated file independently instead of the one-to-all model where a single defragger handles all files, resulting in parallel file defragmentation. We will describe the details of parallel defragmentation as follows.

#### Invoking multiple defraggers.

**Fig 6:**
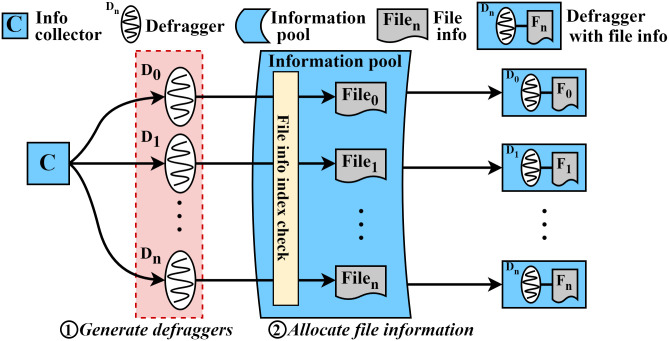
Information collector and multiple defraggers in ScaleDefrag.

To start the parallel defragmentation, as shown in Fig 6, the collector (C) invokes N defraggers (D_0_-D_*n*_) to start working on file defragmentation ①. Each defragger fetches a set of information of a file from the information pool which contains N sets of information of files (file_0_-file_*n*_) ②. Because we allow each defragger to fetch each file in the same order as the collector stores it, the first fetched file will be defragmented first. In this fetch process, we use a global index to indicate the next file that should be assigned to a defragger. Specifically, each defragger atomically claims a unique work item via fetch_and_add() on the global index, which guarantees that no two defraggers receive the same file entry, and makes sure all the defraggers fetch the file information atomically. A defragger stops fetching when the returned index exceeds the pool size, eliminating the need for locks while preventing race conditions.

#### Parallel checking of file fragmentation.

After collecting the target file information, each defragger determines whether the fetched file is fragmented or not by using the file information. First, the defragger checks the size of the file and the number of blocks of the file. If either value is zero, the file is empty and has no content. Thus, it does not require defragmentation. Second, the defragger obtains the number of extents of the file and calculates the optimal number of extents of the file. The optimal number of extents is calculated by dividing the number of blocks of the file by the maximum number of blocks that one extent can have. The file is not fragmented if the current number of extents is less than or equal to the ideal number of extents. When the file is not fragmented, the defragger stops the defragmentation process and fetches the next file information from the information pool. When the file is fragmented, the defragger continues to the next step (i.e., file relocation).

**Fig 7:**
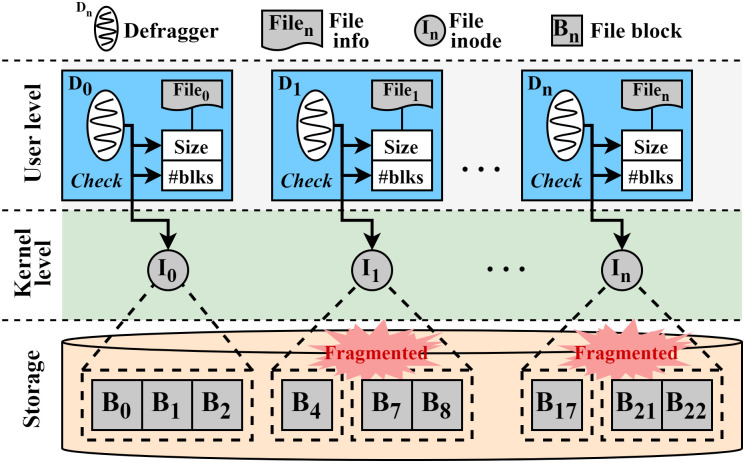
Parallel checking of file fragmentation in ScaleDefrag.

In our scheme, we parallelize this checking operation for the fragmentation as shown in Fig 7. As shown in the figure, there are three defraggers (D_0_, D_1_, and D_*n*_). Each defragger fetches its own file information (file_0_, file_1_, and file_*n*_) with the associated inode (I_0_, I_1_, and I_*n*_). In the case of file_0_, the blocks (B_0_, B_1_, and B_2_) are stored in a single extent so that there is no fragmentation. Thus, D_0_ finishes its defragmentation process for file_0_. In the case of file_1_, the blocks (B_4_, B_7_, and B_8_) are stored in two different non-contiguous extents. It means that file_1_ is fragmented and its blocks can be relocated into a single extent. Therefore, D_1_ will continue to try to relocate blocks of file_1_. Likewise, in the case of file_*n*_, since the blocks are stored in different extents, D_*n*_ will try to relocate file_*n*_. As a result, this strategy realizes one(defragger)-to-one(file) model by enabling each defragger to check the fragmentation of its file simultaneously, thereby reducing the time for fragmentation checking during the defragmentation process.

#### Parallel file relocation.

After identifying fragmented files, each defragger tries to relocate the scattered blocks in its corresponding fragmented file to a contiguous storage space in a parallel manner. Before starting the relocation process, each defragger checks if there is enough contiguous space for the fragmented file. To do this, each defragger creates a temporary file and examines the number of extents (contiguous blocks) within this temporary file. Then, the defragger determines whether to start or stop the relocation process of the fragmented file. If the number of extents of the temporary file is less than that of the fragmented file (more contiguous space), each defragger starts to relocate the file, which means that the newly allocated storage space is less fragmented. Otherwise, the defragger stops the defragmentation process. When starting the relocation process, each defragger reads all the blocks of its fragmented file into the page cache and exchanges the block mapping information between the fragmented file and the temporary file. Specifically, the scattered blocks of the fragmented file are remapped to the temporary file and the newly allocated contiguous blocks are remapped to the fragmented file. When the temporary file is closed, the file and its blocks are automatically reclaimed by the file system. Finally, each defragger writes the blocks to the contiguous space.

**Fig 8:**
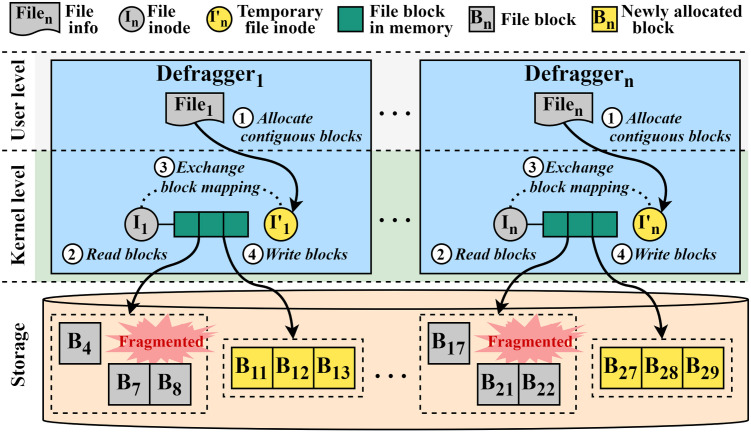
Parallel file relocation in ScaleDefrag.

As shown in Fig 8, for the fragmented file (file_1_), the defragger (D_1_) first allocates a new temporary file inode (I_1_′) with continuous storage space and determines whether to start file relocation or not ①. When starting file relocation, D_1_ reads all the blocks of the fragmented file into memory (i.e., page cache) ②. Once all blocks are read into the memory, D_1_ exchanges the block mapping information between the fragmented file and the temporary file ③ and writes data to the contiguous space ④. Likewise, other defraggers (D_*n*_) perform this defragmentation process for file_*n*_ in parallel. They perform this process continuously until all the files in the information pool are handled. Consequently, since all the defraggers can independently perform this file defragmentation simultaneously, this approach enables multiple I/O operations, thereby increasing the number of parallel I/O requests. This, in turn, can take advantage of the high internal parallelism of SSDs.

### Asynchronous I/O

**Fig 9:**
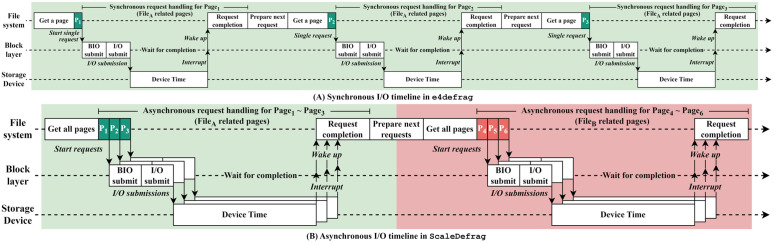
Synchronous and asynchronous defragmentation I/O timeline in e4defrag and ScaleDefrag, respectively. **(A)** Synchronous I/O timeline in e4defrag. The defragger reads one page (P1, P2, P3) at a time, submits a single I/O request, and waits for its completion before preparing and issuing the next request. As a result, the device time for successive pages does not overlap and much of the potential parallelism in the storage device is left unused. **(B)** Asynchronous I/O timeline in ScaleDefrag. The defragger first collects multiple pages related to the same file (P1-P3 and P4-P6), submits their I/O requests, and then waits for completion. This allows the device times of different pages to overlap and exposes higher I/O parallelism, reducing the effective defragmentation time.

As we previously mentioned as in the Synchronous I/O subsection, the existing defragmentation scheme reads scattered blocks of the fragmented file in a synchronous manner. Fig 9A depicts the timeline of the synchronous I/O operation in e4defrag. As shown in the figure, there are three scattered blocks in a file (File_*A*_). In the existing scheme, a single defragger gets a page (page_1_), makes a request with the page (BIO submit), submits the request (I/O submit), waits for the completion of the request, and completes the request. After that, the defragger prepares the next request and performs read operations for other two blocks as described above using page_2_ and page_3_. This means that the defragger cannot issue new requests anymore until the issued request is completed. This strategy limits the read performance within a defragger.

To handle this issue, we adopt an asynchronous I/O approach to perform the I/O operations efficiently during file defragmentation. Although user-level libraries such as Linux libaio (and newer interfaces such as io_uring) provide general-purpose asynchronous I/O APIs, ScaleDefrag does not build on them directly. We implement ScaleDefrag by extending e4defrag and its kernel-side defragmentation path. In this approach, each defragger first issues a batch of read requests for scattered blocks of a fragmented file at once, then waits and completes them. Thus, each defragger can continuously issue the requests of the next blocks regardless of whether the previously issued requests are completed. By doing so, this approach can significantly increase the number of concurrent read requests per defragger. However, higher I/O concurrency due to the asynchronous approach may increase contention (e.g., deeper device queues and more in-flight pages). ScaleDefrag relies on the Linux block layer’s queue-depth limits for backpressure, without introducing an explicit per-defragger throttling mechanism.

Fig 9B shows the proposed asynchronous I/O approach. There are three scattered blocks in one fragmented file (File_*A*_) and in another fragmented file (File_*B*_). Unlike the existing scheme, a defragger gets three pages (page_1_, page_2_, and page_3_), makes three requests, issues the requests at once. The defragger then waits for the three requests and finishes the I/O operation only when all three requests are completed. Subsequently, the defragger gets another file (File_*B*_) and performs I/O operation with pages (page_4_, page_5_, and page_6_) as described above. This asynchronous approach can increase the number of concurrent issued requests per defragger to the storage device. As a result, the total read time during defragmentation can be significantly reduced.

In terms of write operations, we use the same approach as the existing scheme does since the write operations are already performed by the page cache layer asynchronously. For example, the defragger marks the blocks to be relocated as dirty, after then, the page cache layer flushes the dirty blocks to the storage device asynchronously.

#### Correctness and consistency.

Our proposed scheme reduces the execution time of file defragmentation while ensuring correctness and consistency because it satisfies the following properties:

**Correctness:** Each defragger works independently on file defragmentation without interaction with each other. As previously shown in Fig 8, each defragger handles its own file and preallocates the contiguous free space without interfering others. Although a parallel scheme is used, ScaleDefrag ensures the correctness by guaranteeing the independent operation and space among multiple defraggers.

**Consistency:** Although our scheme modifies the mapping information of the fragmented file, our scheme can restore to the previous consistent state even if any failure occurs during the defragmentation process. For example, the data blocks of the file are written to the new continuous space and the original data blocks are still intact until the file relocation is finished. Also, the original metadata information is handled by the journaling system. By doing so, we ensure the data and metadata consistency.

## Discussion and limitation

**Cost and trade-offs:** Defragmentation inherently incurs additional data copying to relocate valid blocks, which increases write traffic and can increase write amplification (WAF), accelerating SSD wear. While ScaleDefrag reduces the defragmentation time by exploiting multi-core parallelism and asynchronous I/O, it does not aim to minimize the total amount of data moved during defragmentation. Thus, the primary trade-off is between faster maintenance (shorter defragmentation time) and the additional write traffic inherent to defragmentation. Although we do not directly measure WAF or quantify the precise impact on SSD lifetime in this study, ScaleDefrag also performs additional data copies during defragmentation as other defragmentation tools do. Quantifying the resulting long-term wear-out impact and designing lifetime-aware policies remain important directions for future work. To reduce this side effect, we can propose a selective policy (e.g., FragPicker [21]). It means that we determine if we defragment a fragmented file or not, or a specific extent of a fragmented file or not according to its degree of fragmentation. For example, FragPicker [21] monitors I/O-related system calls and analyzes the access patterns of applications for each file. After then, it selectively defragments only the blocks that affect application performance (e.g., frequently accessed blocks), thus reducing the data copy caused by defragmentation. FragPicker demonstrates that defragmenting only the frequently accessed blocks can achieve a similar improvement in application performance compared with full defragmentation. As a result, we believe that our scheme can be combined with FragPicker to achieve both exploiting SSD parallelism and reducing the negative effect on SSD lifetime and possible WAF during defragmentation. We leave this combined and cooperative work as future work.

Furthermore, the design of ScaleDefrag introduces CPU and memory overheads. ScaleDefrag reads as much data and metadata about the files as possible into memory to process multiple files concurrently, resulting in high peak memory usage. While running ScaleDefrag on a highly fragmented state, with 16 defraggers, the peak memory consumption on average is 78% (12.53 GB) of the total memory residing in the system, which is 1.13× higher than e4defrag (11.12 GB). The peak memory usage mainly comes from maintaining metadata for many target files and increasing the number of in-flight pages due to concurrent defraggers and asynchronous reads. To reduce the footprint, a straightforward optimization is to adopt incremental, pipelined processing of the file information pool. In detail, we can collect and defragment a bounded set of files, and then release the associated metadata, instead of loading all target file information into DRAM at once. In addition, we can explicitly bound the working set by limiting the number of active defraggers and the number of outstanding requests per defragger, which directly constrains the number of in-flight pages. These methods allow ScaleDefrag to trade peak memory for throughput and to operate on machines with smaller DRAM by selecting a smaller concurrency configuration. Evaluating such memory-constrained settings (e.g., 4–8 GB DRAM) and quantifying the trade-off between performance and memory are important directions for future work. The CPU utilization of ScaleDefrag and e4defrag shows under 33% and 8%, respectively. These overheads stem from our parallel and asynchronous defragmentation, which consumes more cores and memory resources.

**Handling limited space:** ScaleDefrag does not require an entire file to fit in DRAM, as the e4defrag does. The internal page eviction policy of the Linux kernel handles file data through the page cache, allowing defragmentation to proceed incrementally. In detail, the defragmentation tool manages the pages of the target file through page cache. If the dirty page ratio is high or there are not enough free pages in the memory, the Linux memory management evicts the least recently used pages from the page cache. Thus, the pages that have already been processed can be evicted, while the upcoming new pages can be processed continuously. As a result, the Linux page eviction policy allows defragmentation to proceed incrementally without requiring all the pages of the file to reside in memory.

Furthermore, if the free space of the file system is scarce or no sufficiently large contiguous extents can be found, ScaleDefrag follows the same semantics as e4defrag. In detail, the defragmentation process checks the available free space of the file system before allocating contiguous extents for a single file. If there is no free space for the file, the defragmentation tool does not proceed the defragmentation process anymore.

**Logical and physical defragmentation:** ScaleDefrag operates at the logical block level exposed by the file system. Although a flash translation layer (FTL) may subsequently remap physical pages on a future write, retaining logical contiguity still provides benefits. First, logical defragmentation benefits read operations because it maintains logical block contiguity within the file system. As a result, the file system can issue requests for multiple contiguous blocks to the SSD in a single command [21], even if the blocks are fragmented physically within the SSD. Second, logical defragmentation can be utilized without requiring specific hardware or physical layout information, therefore broadly deployable on commercial SSDs without specific hardware modifications or privileged access to the FTL.

**Manycore scalability:** Our main evaluation setup consists of 8 physical cores and a 250 GB SSD, which does not fully represent modern manycore servers. To partially address this limitation, we evaluate ScaleDefrag on a 64-core manycore system with two different SSDs (a consumer SATA SSD and an Intel Optane 900p SSD). The results show that defragmentation time continues to decrease and throughput increases as we scale the number of defragger threads up to 64 cores on both devices, indicating that our parallel and asynchronous design can effectively exploit additional cores. However, we still do not explore even larger-scale deployments such as servers with hundreds of cores or clusters with multiple SSDs per node. Evaluating ScaleDefrag on such systems, and studying its interaction with distributed file systems, remains important future work.

**Future work:** First, while we evaluated ScaleDefrag on up to a 64-core system, it would be valuable to study larger-scale environments (e.g., 100 + cores) and its interaction with distributed file systems or multi-SSD deployments. Second, we plan to integrate ScaleDefrag with selective defragmentation policies such as FragPicker [21] to reduce unnecessary data movement while still exploiting SSD parallelism. Third, we will investigate energy efficiency by measuring power during defragmentation and assessing whether finishing maintenance earlier reduces total energy consumption or shifts energy to short, high-intensity bursts.

## Evaluation

### Experimental setup

For evaluation, we use a machine which has a Intel i9-9900K CPU (16-core with hyperthreading) with 16 GiB memory, unless otherwise mentioned. The machine runs Ubuntu 18.04 with Linux kernel version 5.5.8, and file system is EXT4. For storage, we use a flash-based SSD, Micro CT250MX500 SSD [49] equipped with TLC type NAND flash, four channels, and one die per channel. We run FFSB and filebench [44] workloads (i.e., varmail, fileserver, and OLTP) for our evaluation. We choose varmail and fileserver to emulate common file-service environments (e.g., email-style file creation/update patterns and enterprise/shared storage workloads), and OLTP workload to represent database-style workloads. We additionally use FFSB as a configurable file-system benchmark to generate a distinct fragmentation state and to diversify file sizes and access patterns. Together, these workloads cover a wide range of fragmentation degrees (Table 2) and help evaluate ScaleDefrag under realistic conditions. Specifically, as a fragmentation-generation methodology, we proceed as follows. 1) We run a workload to generate files; the resulting files become fragmented. 2) We then perform defragmentation on this fragmented state.

**Table 2:** Filebench and FFSB workload configurations.

Workloads	Varmail	FFSB	Fileserver	OLTP
**# of files**	1,000,000	2048	1,000,000	1,000
**meandirwidth**	1M	–	20	1,024
**# of threads**	16	16	16	16
**average file size**	32 KB	–	128 KB	10 MB
**total workload size**	16 GB	14 GB	12 GB	9.8 GB

The detailed configuration of these workloads is listed in Table 2. These three workloads generate files with different degrees of fragmentation. e4defrag uses a “fragmentation score” to represent the degree of file fragmentation. The score is calculated by the number of extents before/after defragmentation and the total number of blocks of all files in the file system. The equation is as follows:


$$ratio=\frac{(E_{b}\;-E_{a})*100}{N}$$

$$fragmentation\;score=\left\{ \begin{array}{cc} 80+\frac{ratio}{5}, & ratio>10;\\ 8\times ratio, & otherwise. \end{array}\right.$$

Where:

- *E*_*b*_: is the number of extents before defragmentation.
- *E*_*a*_: is the number of extents after defragmentation.
- *N*: is the number of blocks of all files in the mounted EXT4.

Table 4 shows the fragmentation states of files generated by different workloads. For each evaluation, we create a clean file system by mkfs command and free the page cache before defragmentation. After generating files, we use the -c option of e4defrag to confirm the fragmentation score of the file system and the average extent size. When the number of extents is higher with a certain number of blocks, the fewer blocks are stored in contiguous space. Thus, the higher the score, the more fragmented the file is. The extent size denotes the number of contiguous blocks within an extent. For example, when the blocksize is 4 KB and the extent size is 64 KB, there are 16 contiguous blocks in an extent. Thus, the higher the extent size, the less fragmented the file is. As shown in the table, varmail has the highest degree of file fragmentation (i.e., 84) with an average extent size of 9 KB; fileserver has the second highest fragmentation (i.e., 27) with an average extent size is 64 KB; OLTP has the lowest fragmentation (i.e., 1) with an average extent size of 2.4 MB.

**Fig 10:**
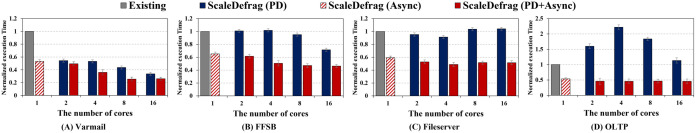
Normalized execution time of the existing and proposed schemes on flash-based SSD. (A) Defragmenting highly fragmented files generated with Varmail workload. (B) Defragmenting moderately fragmented files generated with FFSB workload. (C) Defragmenting less fragmented files generated with Fileserver workload. (D) Defragmenting little fragmented files generated with OLTP workload. The y-axis shows normalized execution time to the e4defrag with one thread (lower is better), and the x-axis shows the number of defragger threads. Across all workloads, ScaleDefrag with both schemes(PD+Async) achieve the shortest defragmentation time.

**Fig 11:**
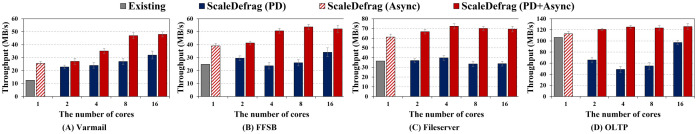
Throughput of the existing and proposed schemes on flash-based SSD (PD: parallel defragmentation, Async: asynchronous I/O). (A) Defragmenting highly fragmented files generated with Varmail workload. (B) Defragmenting moderately fragmented files generated with FFSB workload. (C) Defragmenting less fragmented files generated with Fileserver workload. (D) Defragmenting little fragmented files generated with OLTP workload. The y-axis shows average defragmentation throughput (MB/s) and the x-axis shows the number of defragger threads. In all workloads, ScaleDefrag with both schemes(PD+Async) achieve the highest throughput.

Under these fragmentation states, we evaluate our proposed schemes (i.e., parallel defragmentation and asynchronous I/O) and compare them with the existing scheme (i.e., e4defrag). We vary the number of defragger threads in our proposed scheme. We present the execution time and throughput of defragmentation as shown in Figs 10 and 11, respectively. The execution time is normalized to that of the existing scheme. We run each test ten times and report the average. For each experiment, we recreate the file system with mkfs, drop the Linux page cache, and ensure consistent starting state for the SSD by TRIM in order to mitigate the impact of background garbage collection and wear-leveling. For clarity, we refer to our parallel defragmentation technique as PD, our asynchronous I/O technique as Async, and their combination as PD+Async.

### Experimental results

#### Overall results summary.

As shown in the Table 3, ScaleDefrag outperforms e4defrag mainly by increasing I/O concurrency on commodity SSDs. e4defrag’s synchronous, largely serialized processing limits the number of outstanding requests, leaving SSD internal parallelism underutilized. In contrast, ScaleDefrag combines parallel defragmentation across multiple defraggers with asynchronous issuance of scattered-block reads, which increases the number of concurrent in-flight requests and better matches modern SSD queue-level parallelism. This higher concurrency translates into higher defragmentation throughput and shorter defragmentation time across workloads.

**Table 3:** Baseline comparison summary across workloads. Execution time, defragmentation throughput, and peak memory usage for e4defrag and ScaleDefrag (PD+Async; Parallel and asynchronous).

Workload	Frag. degree	Normalized execution time			Defrag throughput			Peak memory		
		e4defrag	ScaleDefrag	Speedup	e4defrag	ScaleDefrag	Gain	e4defrag	ScaleDefrag	Overhead
Varmail	High	1	0.26	3.84×	12.6 MB/s	48 MB/s	3.8×	11.12 GB	12.53 GB	1.13×
FFSB	Moderate	1	0.46	2.16×	24.8 MB/s	52.2 MB/s	2.16×	10.08 GB	11.32 GB	1.12×
Fileserver	Low/Mod.	1	0.51	1.94×	36.55 MB/s	69.3 MB/s	1.9×	9.72 GB	10.47 GB	1.08×
OLTP	Low	1	0.46	2.19×	106.36 MB/s	126 MB/s	1.18×	9.34 GB	10.39 GB	1.11×

#### Highly fragmented files by varmail.

As shown in Table 4, files generated by varmail workload are highly fragmented with a fragmentation score of 84 and an average extent size of 9 KB. Under this fragmentation state, in terms of execution time, as shown in Fig 10A, the asynchronous I/O scheme (Async) improves the performance by up to 1.88× compared with the existing scheme. This result demonstrates that the asynchronous I/O operations can improve the performance even though a single thread performs the operations. Our parallel defragmentation (PD) scheme improves the performance by up to 1.84×, 1.88×, 2.3×, and 2.96× in the case of 2, 4, 8, and 16 defragger threads, respectively. In the case of varmail, the files are highly fragmented with little spatial locality, which causes a highly random access pattern when reading their blocks. Thus, the parallel defragmentation scheme can be more effective on this varmail workload compared with other workloads (i.e., fileserver, FFSB, and OLTP). The parallel defragmentation with the asynchronous I/O scheme (PD+Async) further reduces the execution time by up to 2×, 2.79×, 3.8×, and 3.85× in the case of 2, 4, 8, and 16 defragger threads, respectively. This means that the asynchronous I/O operation accelerates the parallel defragmentation scheme by issuing multiple requests until there is no any further request and completing them in an asynchronous manner, and so that both schemes exhibit better performance in the case of highly fragmented files.

**Table 4:** Fragmentation states of files evaluation workloads.

Workloads	Varmail	FFSB	Fileserver	OLTP
**Extent size (KB)**	9 KB	23 KB	64 KB	2,480 KB
**# of extents before defrag**	1,656,740	617,517	1,753,750	4,170
**# of extents after defrag**	800,680	417,562	801,260	1,010
**Total # of blocks**	3,727,665	3,578,215	28,060,000	2,585,400
**Fragmentation score**	84	45	27	1

Similarly, Fig 11A shows that the asynchronous I/O scheme improves the average throughput by up to 2× compared with the existing scheme. The parallel defragmentation scheme improves the average throughput by up to 1.8×, 1.9×, 2.14×, and 2.54× when using 2, 4, 8, and 16 defragger threads, respectively. When using both parallel defragmentation and asynchronous I/O schemes, the throughput is further improved by up to 2.15×, 2.78×, 3.73×, and 3.8× in the case of 2, 4, 8, and 16 defragger threads, respectively. This result demonstrates our schemes can increase I/O throughput during the defragmentation process compared with the existing scheme.

#### Moderately fragmented files by FFSB.

The FFSB [50] workload generates files with an average extent size of 23 KB and a fragmentation score of 45, which is moderately fragmented. As shown in the Fig 10B, the asynchronous I/O scheme reduces the total execution time of the defragmentation process by up to 1.54× compared with the existing scheme (e4defrag). The PD scheme reduces the execution time by up to 0.99×, 0.98×, 1.05×, and 1.4× in the case of 2, 4, 8, and 16 defragger threads, respectively. Also, by utilizing parallel defragmentation with asynchronous I/O scheme (PD+Async), the execution time reduces by up to 1.62×, 1.98×, 2.13×, and 2.16× as the number of defragger increases.

Fig 11B shows the average throughput during the defragmentation process. The throughput of defragmenting files in a moderately fragmented state is improved, similarly to the execution time. The parallel defragmentation scheme increases the average throughput by up to 1.38× compared with the serialized scheme. The asynchronous I/O scheme with the parallel defragmentation scheme improves the throughput by up to 2.16× compared with the existing scheme. This represents ScaleDefrag can increase SSD throughput during defragmentation even in the case of moderately fragmented files compared with e4defrag.

Unlike highly fragmented files, in the case of moderately fragmented files, the parallel defragmentation scheme has little effect or rather decreases the performance. The reason is that the files generated by FFSB have a relatively larger extent size (i.e., 23 KB) compared with the case of varmail. This means that the number of contiguous blocks in an extent increases. Thus, since the contiguous blocks can be processed in an I/O request, the total number of I/O requests to be transferred to the storage device decreases, therefore, the effectiveness of the parallel defragmentation scheme can be reduced.

Meanwhile, even though only the parallel defragmentation scheme has a minor effect on large extent sized workload, the scheme accelerates the asynchronous I/O scheme and so that further improves the I/O performance. Consequently, even in the case of moderately fragmented files, ScaleDefrag can significantly reduce the defragmentation time compared with e4defrag.

#### Less fragmented files by fileserver.

The fragmentation score of files generated by fileserver workload is 27 and the average extent size is 64 KB. This denotes that the degree of fragmentation by fileserver is less than that by that of varmail and FFSB. Fig 10C shows that the asynchronous I/O scheme (Async) reduces the execution time by up to 1.68× compared with the existing scheme. The parallel defragmentation (PD) scheme reduces the execution time by up to 5%, 9.5%, −3%, and −3.8% in the case of 2, 4, 8, and 16 defragger threads. Unlike highly and moderately fragmented files, the parallel defragmentation scheme has little effect or rather decreases the performance in the case of less fragmented files. The reason is that the files generated by fileserver have a relatively large extent size (i.e., 64 KB) compared with the cases of varmail and FFSB.

Meanwhile, even though only the parallel defragmentation scheme is a little effective on a large extent sized workload, the parallel defragmentation scheme with the asynchronous I/O scheme (PD+Async) shows the highest performance. It is because the parallel defragmentation scheme can accelerate asynchronous I/O scheme. As shown in Fig 10C, the parallel defragmentation scheme with asynchronous I/O scheme reduces the execution time by up to 1.89×, 1.98×, 1.96×, and 1.96× in the case of 2, 4, 8, and 16 defragger threads. The results of throughput show the similar improvement as those of the execution time as shown in Fig 11C.

#### Little fragmented files by OLTP.

The fragmentation score of files generated by OLTP is only 1, and the average extent size is 2.4 MB. Since the average extent size of OLTP is much larger than that of fileserver, as shown in Fig 10D, the execution time of the parallel defragmentation scheme is up to 2.22× longer than that of the existing scheme. Meanwhile, the asynchronous I/O scheme (Async) reduces the execution time of existing scheme by up to 1.87×. When using both parallel defragmentation and asynchronous I/O (PD+Async) schemes, their execution time reduces by up to 2.19× compared with only asynchronous I/O. In Fig 11D, as expected, the parallel defragmentation scheme harms the throughput due to the large extent size of OLTP. Meanwhile, both schemes improve the performance by up to 33% compared with only the asynchronous I/O scheme. Consequently, through our results, the extent size is an important factor in our schemes. As the extent size is smaller, the parallel defragmentation scheme is more effective. On the other hand, when the extent size is higher, the asynchronous scheme is more effective. Also, combining both two schemes provides a higher performance compared with each scheme.

#### Co-running impact on application with defragmentation schemes.

**Fig 12:**
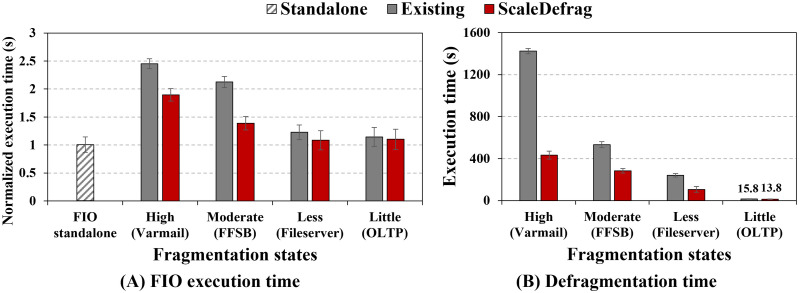
Impact on co-running application with defragmentation schemes (Standalone: running FIO alone). (A) Normalized FIO execution time when running alone (Standalone) and when co-running with the e4defrag or ScaleDefrag under different fragmentation states. (B) Defragmentation time of the e4defrag and ScaleDefrag for the same states. ScaleDefrag both shortens defragmentation time and reduces the slowdown of the co-running application compared with the existing tool.

To quantify the impact on co-running user application with defragmentation schemes, we execute the FIO micro-benchmark concurrently with each defragmentation scheme. FIO is configured as 4 threads, 4 KB sequential write workload whose standalone throughput reaches up to 354 MB/s on our test bed. After first creating four fragmentation states with the varmail, FFSB, fileserver, and OLTP workloads, we co-run FIO with either e4defrag or ScaleDefrag and measure the average FIO execution time as well as the time required to finish defragmentation. The FIO execution times for each fragmentation state are normalized against the execution time of standalone FIO, which is set to 1. As shown in Fig 12A, running FIO together with e4defrag increases the execution time of FIO by up to 2.45×, 2.12×, 1.22×, and 1.14× in the high, moderate, less, and little fragmented cases, respectively. In the case of running FIO with ScaleDefrag, the execution time of FIO is increased by up to 1.89×, 1.39×, 1.08×, and 1.10× in the high, moderate, less, and little fragmented cases, respectively. As a result, ScaleDefrag reduces the FIO execution time than e4defrag by up to 1.53×, in the case of moderately fragmented files.

This is because, as shown in Fig 12B, ScaleDefrag finishes up to 3.29× faster than e4defrag for the highly fragmented files while running alongside FIO. In addition, ScaleDefrag reduces defragmentation time 1.88×, 2.26×, and 1.15× in the case of moderate, less, and little fragmented states, respectively. Although both schemes hinder user application execution while defragmentation processes are active, ScaleDefrag minimizes the penalty on the user application and enables the system to use contiguous blocks sooner. Furthermore, our fast defragmentation quickly releases system resources by shortening defragmentation completion, in the end, the process interferes less with user requests compared with the e4defrag.

#### Core scalability on a manycore system with different SSDs.

To examine how well ScaleDefrag scales beyond our 8-core testbed, we additionally evaluate defragmentation schemes on a 64-core manycore machine with two different SSDs: a CT250MX500 SATA SSD and an Intel Optane 900p NVMe SSD. In this experiment, we compare the e4defrag and ScaleDefrag while increasing the number of defragger threads from 1 to 64, while creating highly fragmented files by varmail workload.

**Fig 13:**
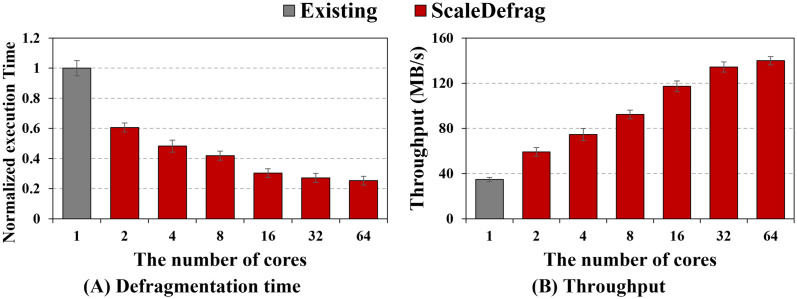
Core scalability of ScaleDefrag on a manycore machine with a CT250MX500 SSD. (A) Normalized defragmentation time of the e4defrag and ScaleDefrag as the number of defragger increases from 1 to 64 (lower is better). (B) Defragmentation throughput (MB/s) under the same settings. ScaleDefrag continuously reduces execution time and increases throughput as more cores are used.

Fig 13 shows the normalized defragmentation time and throughput on the CT250MX500 SSD. As shown in Fig 13A, we normalize the execution time of ScaleDefrag to the execution time of the e4defrag with a single thread. As the number of cores increases, ScaleDefrag gradually reduces the execution time and reaches 3.95× of the baseline at 64 cores. In detail, ScaleDefrag reduces the execution time by up to 1.65×, 2.07×, 2.38×, 3.30×, and 3.68× in the case of 2, 4, 8, 16, 32 defragger threads. The Fig 13B shows that the throughput of ScaleDefrag also scales well as we add more defraggers, achieving 4.02× higher throughput at 64 cores compared with the e4defrag. Specifically, ScaleDefrag improves the defragmentation throughput 1.70×, 2.14×, 2.65×, 3.37×, 3.86×, and 4.02× as the number of defragger increases. These results indicate that ScaleDefrag can effectively exploit additional cores on a SATA SSD.

**Fig 14:**
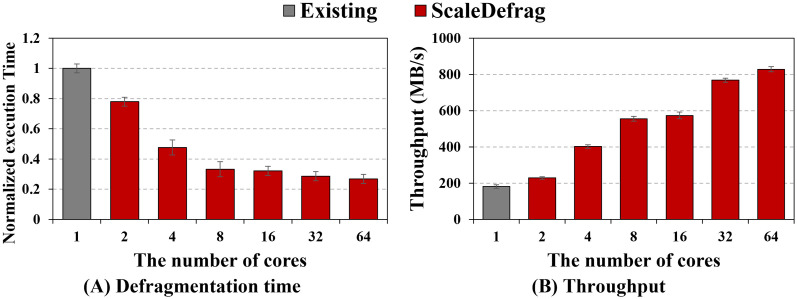
Core scalability of ScaleDefrag on a manycore machine with an Intel Optane 900p SSD. (A) Normalized defragmentation time of the existing tool and ScaleDefrag as the number of defragger increases from 1 to 64 (lower is better). (B) Defragmentation throughput (MB/s) under the same settings. ScaleDefrag continually reduces execution time and increases throughput as more cores are used, reaching 4.57× higher throughput than the e4defrag at 64 cores.

Similarly, Fig 14 presents the same experiment on the Intel Optane 900p SSD. As shown in the Fig 14A, ScaleDefrag again reduces the execution time as the number of cores increases by up to 1.28×, 2.10×, 3.01×, 3.11×, 3.49×, and 3.73×, in the case of 2, 4, 8, 16, 32, 64 defraggers.

In Fig 14B, the throughput of ScaleDefrag grows from less than 181.35 MB/s with a single core to 828.32 MB/s with 64 cores, providing 4.57× higher throughput than the existing e4defrag. Compared with the SATA SSD (e.g., CT250MX500), the NVMe SSD (e.g., Intel Optane 900p) exposes higher internal parallelism, so ScaleDefrag can achieve a larger throughput while still benefiting from additional cores.

Overall, the results demonstrate that our parallel and asynchronous design scales well on a manycore machine and that the benefits of ScaleDefrag persist across different types of SSDs.

### Performance breakdown

To provide a deeper understanding of the effect of ScaleDefrag, we conduct a performance breakdown in terms of execution time from the user level to the kernel level. Table 5 shows the breakdown by measuring the execution time of major functions by using a nano-second-wise time function for high accuracy in the case of varmail workload.

**Table 5:** Performance breakdown of e4defrag and ScaleDefrag (PD: parallel defragmentation, Async: asynchronous I/O).

Defragmentation	Average	Total (Average)	User-level		Kernel-level		
schemes	throughput	execution time	call_defrag()	others	mext_page_mkuptodate()	wait_on_buffer()	others
**e4defrag**	12.6 MB/s	586.61 s	297.19 s	289.42 s	1.80 s	235.68 s	59.71 s
**ScaleDefrag (Async)**	25.6 MB/s	350.61 s	127.44 s	223.17 s	4.39 s	90.41 s	32.65 s
**ScaleDefrag (PD)**	32.2 MB/s	196.29 s	130.36 s	65.93 s	0.35 s	63.49 s	66.52 s
**ScaleDefrag (PD+Async)**	48.0 MB/s	182.48 s	110.76 s	71.72 s	1.32 s	16.03 s	93.41 s

At the user level, a main function (i.e., call_defrag()) is called to start the actual defragmentation process which performs migration of the block extents and exchange metadata between the fragmented file and the temporary file inside the kernel level. Thus, the execution time of call_defrag() is equal to the sum of execution times of kernel level functions (i.e., mext_page_mkuptodate(), wait_on_buffer(), others). Specifically, inside call_defrag(), the process enters the kernel level by issuing an ioctl system call. Then, the process reads the target blocks via mext_page_mkuptodate() and waits for their I/O completion via wait_on_buffer(), which is performed in a synchronous and serialized manner.

As shown in the table, in the case of e4defrag, the mext_page_mkuptodate() function only takes 1.80 seconds, which accounts for nearly 0.006% of total execution time in the kernel level. Meanwhile, the wait_on_buffer() takes 235.68 seconds, which accounts for 81.4% of total execution time at the kernel level. The other parts take 59.71 seconds including different operations such as mapping between the block and the page, and swapping the extents between two inodes.

ScaleDefrag(Async) which is only the asynchronous approach successfully reduces the total execution time by up to 40.23% compared with e4defrag by reducing the execution time of wait_on_buffer() by up to 61.64% compared with e4defrag. However, there is still a limitation of low parallelism by the single thread. Meanwhile, ScaleDefrag(PD) with only the parallel defragmentation approach reduces the total execution time by up to 66.54% compared with the serialized approach. However, ScaleDefrag(PD) still has the overhead from wait_on_buffer().

Finally, ScaleDefrag(PD+Async) shows the lowest total execution time since it performs both parallel and asynchronous I/O operations. Specifically, ScaleDefrag(PD+Async) reduces the execution time in mext_page_mkuptodate() and wait_on_buffer() to 1.32 and 16.03 seconds, respectively, resulting in the highest throughput. Consequently, this result demonstrates that both approaches are required in the defragmentation process.

## Related work

**SSD internal parallelism:** Chen et al. [34] and Saif et al. [35] investigate how I/O access patterns can affect the performance of SSDs. They vary I/O characteristics such as the size of requests, the number of concurrent requests, etc. The results show that increasing the size of requests and increasing the number of requests are two rules to better utilize the SSD internal parallelism, thus achieving better I/O performance. Likewise, we exploit this SSD internal parallelism to accelerate the process of file defragmentation.

**Defragmentation techniques:** Several studies report the impact of file fragmentation on SSD performance [17,24,25,30]. The widely used online defragmentation tool e4defrag (e4defrag) improves logical contiguity, but its serialized and synchronous execution limits multi-core parallelism and I/O concurrency. Park et al. [27] present a cleaning scheme of the log-structured file system (LFS) on SSDs to address the file fragmentation problem. The cleaning scheme reorders the valid data blocks belonging to a victim segment based on the inode numbers during the cleaning process of LFS. FragPicker [21] minimizes the amount of I/Os induced by defragmentation by only migrating necessary blocks (e.g., frequently accessed blocks) of files. To do this, it monitors the I/O related system calls and finds the frequently accessed blocks of files. Janusd [23] introduces a decoupled defragger by utilizing flash storage’s internal logical block to the physical block mapping table. It remaps the logical block addresses (LBAs) of the logically fragmented files with the FTL mapping table. To utilize the FTL mapping table, this scheme requires a special SSD to expose the FTL to the host side. Thus, this scheme can be difficult to use in a system with commercial SSDs. Jun et al. [43] propose an NVMe command extension combined with a page-to-die allocation algorithm designed to ensure that contiguous blocks always land on successive dies, even in the face of file fragmentation or overwrites. These studies [23,27,43] motivate efforts to mitigate fragmentation, but many require device support or focus on reducing migration traffic rather than accelerating the defragmentation process.

To the best of our knowledge, prior approaches either focus on minimizing defragmentation induced I/O traffic or require non-commodity support, but they do not jointly leverage multi-core parallel defragmentation and asynchronous I/O for commodity SSDs. ScaleDefrag fills this gap by combining parallel defragmentation across multiple defraggers with asynchronous issuance of scattered-block reads on off-the-shelf SSDs. Additionally, in our previous work [38], we propose the information collector and parallel defragmentation. In this article, we extend that design by incorporating an asynchronous I/O technique and present ScaleDefrag.

**Scalable file systems:** Min et al. [51] conduct extensive analysis on many-core scalability of five different file systems by using FxMark benchmark suite. They show the scalability bottlenecks from inherent designs of the file system core designs. Hare [52] is a scalable file system that supports multi-core system without cache-coherent shared memory. MAX [53] presents a multicore-friendly log-structured file system to scale in-memory data structure access while delivering concurrency-friendly on-disk format. ScaleFS [54] decouples the in-memory file system from the on-disk file system to achieve core scalability. Also, ScaleFS supports consistency by using operation log which timestamps the logged operations at their each linearization point. SpanFS [55] adopts the decentralized design to resolve the scalability issue on file systems. SpanFS [55] consists of a collection of micro file system services to scale file systems to many cores. Our study is in line with these studies [51–55] in terms of improving the multi-core scalability. In contrast, we focus on scaling the defragmentation process on multi-cores.

## Conclusion

Due to the serialized and synchronous I/O approach, the existing file defragmentation tool (i.e., e4defrag) can be time-consuming even with flash-based SSD. To reduce the defragmentation time, this article presents an efficient scalable file defragmentation tool, ScaleDefrag. ScaleDefrag performs the asynchronous and parallel file defragmentation process to exploit the high parallelism of modern hardware (i.e., multi-cores and multi-channel SSDs). Specifically, in our ScaleDefrag, we adopt a one(defragger)-to-one(file) model which allows multiple defraggers to perform the defragmentation and I/O operations for multiple files in parallel. Furthermore, we devise an asynchronous I/O approach which enables each defragger to issue multiple requests for the scattered blocks and complete them asynchronously. Through evaluation, we demonstrate that ScaleDefrag reduces the execution time by up to 3.8× compared with e4defrag.
